# Open, Laparoscopic, and Robotic Approaches to Treat Colorectal Cancer: A Comprehensive Review of Literature

**DOI:** 10.7759/cureus.38956

**Published:** 2023-05-13

**Authors:** Jessica Vilsan, Sai Aditya Maddineni, Nayab Ahsan, Midhun Mathew, Nikhila Chilakuri, Nilay Yadav, Eduardo J Munoz, Muhammad A Nadeem, Kiran Abbas, Waleed Razzaq, Zain U Abdin, Moiz Ahmed

**Affiliations:** 1 Surgery, Dr Bhausaheb Sardesai Talegaon Rural Hospital, Pune, IND; 2 Surgery, Avalon University School of Medicine, Willemstad, CUW; 3 Surgery, UChicago Medicine AdventHealth GlenOaks, Glen Oaks, USA; 4 Internal Medicine, Quaid-e-Azam Medical College, Bahawalpur, PAK; 5 Internal Medicine, Pennsylvania Hospital, Philadelphia, USA; 6 Medicine, Avalon University School of Medicine, Willemstad, CUW; 7 General Physician, Rama Medical College, Kanpur, IND; 8 Surgery, Montemorelos University, Montemorelos, MEX; 9 Medicine and Surgery, Shifa International Hospital, Islamabad, PAK; 10 Community Health Sciences, Aga Khan University, Karachi, PAK; 11 Internal Medicine, Services Hospital Lahore, Lahore, PAK; 12 Medicine, District Headquarter Hospital, Faisalabad, PAK; 13 Cardiology, National Institute of Cardiovascular Diseases, Karachi, PAK

**Keywords:** natural orifice transluminal endoscopic surgery (notes), single-incision laparoscopic surgery, robotic surgery, laparoscopic surgery, surgical approaches, colorectal cancer

## Abstract

Surgery is usually required to treat colorectal cancer (CRC). Medical technology has advanced, providing various approaches to tackle this disease. Different surgeries are available, such as laparoscopic surgery, single-incision laparoscopic surgery, natural orifice transluminal endoscopic surgery, and robotic surgery. Laparoscopic surgery has several benefits including reduced blood loss and shorter recovery time. It can also improve lung function and minimize complications. However, it requires more time to perform and has a higher risk of complications during the procedure. Robotic surgery provides a three-dimensional view of the surgical area allowing for greater precision in rectal surgeries and access to difficult-to-reach pelvic regions. This method utilizes robotics technology which reduces surgical time and speeds up recovery for patients. There are various surgical options available for treating CRC; however, laparoscopic surgery and robotic surgery offer unique advantages despite their own drawbacks. As technology continues to evolve, medical techniques will continue improving existing methods while providing new options resulting in better outcomes for patients. Compared to laparoscopy, robotic surgery has a lower rate of operative conversions and a shorter learning curve. However, it also has some drawbacks, such as a longer docking time, lack of tactile sensation, and higher cost. Therefore, the choice of surgical method should depend on patient characteristics, surgeon preference and expertise, and available resources. Currently, specialized centers offer robotic surgeries which are more expensive and take longer compared to open and laparoscopic approaches. Nonetheless, they are considered safe and feasible when compared to traditional surgery. Short-term outcomes for robotic surgeries are better, while long-term postoperative complication rates remain similar. However, there is a need for additional well-defined randomized control trials conducted across multiple centers to validate the use of robotic surgery over open and laparoscopic approaches. Improving patient care and outcomes is the objective of this comprehensive literature overview on surgical approaches for CRC.

## Introduction and background

Colon cancer is a type of cancer that can be avoided and is frequently diagnosed in the United States among both men and women, making it the third most common cancer. Colonoscopy, which is easily obtainable for everyone, can detect colon cancer at an early or later stage [1]. Studies have shown that colorectal cancer (CRC) ranks as the fourth most widespread cancer in men and the fifth most prevalent cancer in women. Concerning developments in CRC incidence should be considered when developing health policies in this area. In addition to better socioeconomic situations, the advancement of civilization and economic growth also leads to the "westernization" of lifestyles or changes in food patterns. Obesity and overweight are linked to a higher risk of many modern diseases [2]. The prognosis of CRC in men has been observed to be negatively impacted by visceral obesity. Approximately 25% of the hereditary propensity is genetic. The emergence and progression of cancer are profoundly influenced by variables that have an impact on the duration of life, including behavioral aspects such as smoking, obesity, and physical activity, as well as socioeconomic factors such as education, income, and governmental allocation of resources to healthcare. In devising strategies aimed at averting or managing cancer, it is crucial to take into account the levels of life expectancy [3].

Despite the fact that colon and rectal cancers can be classified separately based on their origin, they share numerous biological and clinical characteristics that often lead to their conflation. It is imperative to recognize that CRC may arise in either the colon or rectum. Adenocarcinomas account for up to 95% of CRC cases, followed by carcinoid tumors, gastrointestinal stromal tumors, lymphomas, and sarcomas. The majority of tumor glands (over 95%) form in well-differentiated adenocarcinomas which is significantly higher than moderately and poorly differentiated adenocarcinomas at 50-95% and 50%, respectively. Moderately differentiated adenocarcinoma is the most commonly diagnosed type in clinical practice (70%), while poorly or highly differentiated adenocarcinomas represent 20% and 10% of cases, respectively [4]. The proximal colon accounts for approximately 41% of all CRC cases, while the distal colon and rectum account for around 22% and 28%, respectively. However, there may be variations in the location based on age and gender [5]. Colonoscopy is an easily accessible method to diagnose this cancer at either an early or later stage [1].

CRC is associated with various risk factors which include being over 50 years old, suffering from long-term constipation, smoking, infection by the human papillomavirus, excessive alcohol consumption, genetic syndromes such as hereditary non-polyposis CRC or Lynch syndrome and Muir-Torre syndrome, Cowden syndrome, and Juvenile polyposis syndrome. Other contributing factors are low levels of physical activity, inadequate intake of fiber-rich foods, and high fat and red meat consumption. Additionally, conditions such as acromegaly and renal transplantation with immunosuppression can increase the risk of this type of cancer. Furthermore, familial adenomatous polyposis (FAP), hamartomatous polyposis syndromes such as Peutz-Jeghers syndrome, and long-term androgen deprivation therapy have also been identified as potential risk factors for CRC [1,2]. Effective treatment for CRC is required because, for CRC survivors, the risk of increased chronic diarrhea and recurrence of cancer is observed [6]. Additionally, nutrition has several interaction mechanisms that can affect the development of CRC, such as effects on immunological reactivity, inflammation, and intestinal flora. Dietary variables accounted for 3.48 million deaths among women in 2019 and 4.47 million deaths among men, making them the second and third major causes of death, respectively. According to reports, modifiable risk factors were responsible for 47% of CRC cases in the United States and 45% of cases in the United Kingdom [7].

Colon cancer is an ideal candidate for screening due to the identification of adenomatous polyp as a distinct premalignant lesion and the favorable outcomes achieved by early detection and subsequent treatment. The primary objective of screening is to identify 90% of sporadic cases of CRC [8]. Approximately 11% of CRC cases result in full recovery among individuals who are younger than 50 years old. Furthermore, the incidence of this illness has been observed to increase annually by approximately 1-2%. This trend is alarming, prompting the United States Preventive Services Task Force to release new colon cancer screening recommendations in 2021. As a result, it is now advised that individuals with an average risk of colon cancer begin screening at the age of 45 [9]. Various geographical regions across the globe are guided by distinctive societies that offer authorized directives for the management of colon cancer. The foremost eminent organizations include the National Comprehensive Cancer Network, the European Society of Medical Oncology, and the Japanese Society for Cancer of the Colon and Rectum [10,11].

The primary way of treating curable CRC is through surgery. While neoadjuvant chemotherapy isn't the preferred first line of treatment, it's becoming more common for locally advanced colon cancer before surgery. This method has been successful in reducing the size of the tumor and improving negative resection rates [12]. Adjuvant chemotherapy is primarily administered subsequent to surgical intervention in patients afflicted with stage III and stage IV colon cancer [13]. The conventional mode of treatment for the past century has involved an open colectomy; however, this procedure has proven to be quite traumatic, with a significant increase in morbidity rate and longer recovery period. Laparoscopic techniques were used for the first colectomy in 1991, and patients have had a faster recovery, less operative pain, and shorter hospital stay [14]. Over the past two decades, laparoscopic surgeries have exhibited transient advantages. However, their long-term effectiveness has been compared to that of open approaches [14-16]. The duration of adjuvant chemotherapy remains unsettled due to inconsistent trends observed for overall survival and disease-free survival in multiple clinical trials [17]. Circulating tumor DNA (ctDNA) is present in the bloodstream of colon cancer patients as a result of necrosis in neoplastic cells. Various studies have demonstrated that ctDNA serves as a valuable diagnostic marker with favorable clinical efficacy. The absence of ctDNA has been linked with better treatment outcomes while its presence after treatment has led to inferior outcomes according to multiple studies [10-18]. This review will concentrate on the surgical management of colon cancer using open, laparoscopic, and robotic approaches for comparative purposes.

## Review

Epidemiology of CRC

Over the past five years, CRC has become one of the most common types of cancer, and it continues to be one of the primary causes of cancer-related deaths [17,18]. According to GLOBOCAN 2020 data, there were an estimated 865,630 new cases in females and 1,065,960 new cases in males of all ages. This corresponds to about 9.4% and 10.6% of all cancer cases for females and males, respectively [19]. In that same year, CRC accounted for approximately 1.93 million incidences and resulted in around 0.94 million deaths worldwide. The incidence and mortality rates for CRC vary across countries due to various factors involved in diagnosis and management [5,20]. Generally speaking though, developed countries have a higher prevalence of this disease than developing ones; however, with the increasing westernization of diet and lifestyle in developing nations like India or Brazil [21], experts predict an uptick in global frequency. In fact, as recently as last year (2020), countries like Russia, India, Germany, Brazil, the United Kingdom, Italy, and France, among others, had some of the highest reported cases globally [19]. In the United States today, about one out of every 23 males or 25 females will receive a diagnosis [3,22]. With regards to gender-based statistics, according to recent data (2018-2019), men are more susceptible than women when it comes to getting diagnosed with CRC, especially so: Among all cancers surveyed by medical professionals internationally, CRC was found to be the most prevalent among men compared with women. This excludes non-melanoma skin cancer diagnoses [4]. It is expected that these figures will only rise over time with an estimated increase rate close to or at around 63% by 2,040 alone which signals a pressing need for more research into both prevention measures as well as better detection methods [8,23].

Risk factors of CRC

CRC is the third most common cancer worldwide, and understanding the pattern of distribution and factors that may correlate to the occurrence of the disease is necessary and would form the crux of early detection in programs. The factors that have been associated with the disease can be non-modifiable and modifiable.

Non-modifiable risk factors

Gender

Women, although 1.5 times less likely to suffer from CRC than men [24], are prone to the more aggressive form of right-sided colon cancer [25]. The risk of developing CRC shows a huge disparity among the sexes with increasing age, a 2.5-fold increase in males of 50-59 years and a fivefold increase in males of 60-69 years when compared to females of the same age groups, respectively, in comparison to males and females of age 40-49 years which did not have statistically significant differences [26].

CRC is classically seen in the elderly, but over the recent past, as per SEER statistics, the incidence of CRC among the young population (early onset CRC < 50 years) is rising which may be the consequence of a rise in sedentary lifestyle and obesity among the young [27]. This early-onset CRC (EoCRC) has been an issue of concern ever since its discovery since most of the screening programs are catered toward the elderly, and by the time EoCRC is detected, the disease had progressed to its later stages [27].

Even though the majority of cases of CRC are seen in old age, with around 10% seen in people of age <50 years, these cases are classified as EoCRC [28]. Since CRC screening programs are catered toward the elderly, the majority of EoCRC cases are diagnosed at later stages.

Genetic

Colonic polyps in CRC can be classified into polyposis and non-polyposis syndromes based on their quantity and histology. FAP with germline APC gene mutations is one of the polyposis syndromes. Despite the fact that FAP has an autosomal dominant mode of inheritance, the majority of cases that have been identified are caused by spontaneous de novo APC gene mutations. An autosomal recessive polyposis condition called MUTYH-associated polyposis (MAP) is brought on by germline mutations in the MUTYH gene. Germline mutations in polymerase epsilon (POLE) and delta (POLD1) are linked to another condition known as polymerase proofreading-associated polyposis (PPAP) [28].

Lynch syndrome, which is caused by mutations in the MLH1, MSH2, MSH6, EpCAM, and PMS2 genes, continues to be the most prevalent hereditary condition linked to CRC. There's about a 20% chance for people with Lynch syndrome to develop CRC by the age of 50 and a 50% chance for CRC to develop by the age of 70 [28]. This syndrome tends to present early in the young and quickly develops to an advanced stage [29].

Modifiable risk factors

Dietary Habits

A 3% rise in risk is seen to be associated with every 5 kg rise in weight [30]. Diet has become such a significant factor that red meat has been labeled as "probably carcinogenic," whereas processed meat is labeled as "carcinogenic" by the International Agency for Research on Cancer. The reason for said carcinogenesis being high temperature cooking and curing [31]. Fiber present in fruits and vegetables has a protective role since it tends to shorten the exposure of potential carcinogens in food by allowing for quicker intestinal transit time [32].

Smoking and Alcohol Intake

Smoking, both past and current smokers, is associated with a poorer prognosis in CRC, and smoking cessation proves to be a factor for better survival rates [33]. Similarly, it has been observed that alcohol intake is associated with the development of CRC, especially in heavy drinkers [34].

Clinical manifestations

In primary care, CRC is one of the most frequently missed diagnoses. Asymptomatic individuals are discovered by routine screening [35]. Typical symptoms include rectal bleeding, abdominal pain, and anemia due to fecal occult blood. Presenting signs and symptoms are associated with the location of the tumor. Proximal tumors mostly present with marked anemia, whereas distal tumors mostly present with rectal bleeding and abdominal mass [36,37]. The lack of bleeding and change in bowel habits in proximal cancers lead to delayed diagnosis of proximal cancer. In contrast, rectal bleeding in distal cancers makes the diagnosis earlier [38]. Other nonspecific symptoms include a distended abdomen, lower abdominal pain, constipation, blotting, diarrhea, lack of appetite, unusual tiredness, and involuntary weight loss. Abdominal pain is usually in cases of complications like perforation or fistula formation. Weight loss is solemnly present alone without other rectal bleeding or obstruction symptoms [39]. Older age and Iron deficiency anemia are crucial elements of right-sided CRC. Abdominal distention, nausea, and vomiting are less prevalent presenting symptoms but may be signs of intrinsic obstruction. Obstructive symptoms are more familiar with tumors that encircle the intestine, leading to the so-called apple-core description, most prominently seen in barium enema. This procedure is now rarely utilized [40].

Some studies show no significant association of rectal cancer with weight loss, constipation, diarrhea, and abdominal pain, while others show a strong association [41,42]. Specificities of rectal bleeding and fecal occult blood are 99% and 55.7%, respectively, 69.2% of distal colon cancers, and 38.9% of proximal cancer [42-47]. Specificities for unpaired symptoms are usually low, whereas paired symptoms have relatively high specificities.

Management of CRC

Management of stage 1 cancer is local resection with lymph node dissection [43-49]. The overall survival rate is high when the tumor is at an early stage. Those presenting with abscess formation should first be treated for abscess and then surgical resection of the local tumor [49]. Laparoscopic right hemicolectomy is well-known for right-sided tumors. Extracorporeal anastomosis (EC) is the gold standard, whereas intracorporeal anastomosis is equally effective. Complete mesocolic excision is also effective in right-sided tumors. Still, surgery gets prolonged due to the difficult dissection of vessels [49,50]. Most patients are metastatic at the time of presentation. Overall prognosis is poor in these patients leading to a decrease in the five-year survival rate. In these patients, the usual approach is adjuvant therapy. Chemotherapy or radiotherapy is done before or after primary resection [48].

Surgical approach for CRC

Table 1 illustrates a comparison of the three main surgical approaches for the management of CRC. In the following sections, we have discussed those approaches in detail.

**Table 1:** Comparison between open, laparoscopic, and robotic surgery for the management of CRC

Procedure	Advantages	Disadvantages
Open surgery		
1	Better approach to manage complications such as obstruction, perforation, and hemorrhage in CRC [68].	Open surgery typically requires larger incisions than minimally invasive techniques, which can result in more tissue damage, scarring, and longer healing times. A larger incision size leaves a permanent scar at the site of the incision. Incision size correlates with the intensity of postoperative pain at the site [69].
2	Direct visualization: Open surgery provides a direct line of sight to the surgical site, allowing the surgeon to see and assess the area in real time. This can be particularly useful in complex surgeries or cases where there are unexpected findings [69].	Open surgery typically requires recovery from open surgery which can take longer than minimally invasive techniques due to the larger incisions, increased pain, and longer hospital stay in regards to managing wound health and postoperative surgical site infections [68,69].
3	Open surgery allows for greater access to the surgical site, which can be important in cases where there are large tumors, extensive scarring, or other factors that make it difficult to reach the area with minimally invasive techniques.	Because open surgery involves larger incisions and more tissue manipulation, there is a higher risk of infection compared to minimally invasive techniques.
4	Open surgeries are preferred in patients with a history of multiple surgeries as extensive intra-abdominal adhesions limit access in conventional laparoscopic surgery [70].	Open surgery can carry a higher risk of complications such as blood clots, pneumonia, and other surgical site infections. Longer duration of postoperative ileus even with early ambulation as compared to laparoscopic surgeries [71].
5	Surgeons performing open surgery can feel the tissue and organs they are operating on, which can provide valuable tactile feedback to guide their movements and ensure accuracy.	Open surgery can result in greater blood loss during the procedure, which may require blood transfusions or other interventions to manage.
Laparoscopic		
1	Laparoscopic surgery involves making smaller incisions than open surgery, which results in less tissue damage, reduced scarring, faster healing times, and better cosmetic outcome [72].	The surgical field is converted to a two-dimensional image on a monitor, devoid of a three-dimensional view compared to open or robotic surgery [73].
2	Patients who undergo laparoscopic surgery typically experience a faster recovery time due to the smaller incisions and reduced tissue damage, resulting in less postoperative pain which allows for early mobilization, minimal use of analgesics, and shorter hospital stay.	Difficulty in controlling intraoperative hemorrhage and inadvertent injury to adjacent structures(Injury to the left ureter and bladder in sigmoid colectomy) [74].
3	Laparoscopic surgery uses a camera to provide high-quality, magnified images of the surgical site, which can improve surgical precision and reduce the risk of complications [71].	Pneumoperitoneum used in laparoscopic surgeries can cause shoulder pain due to referred pain from the diaphragm, which can be particularly bothersome in patients who are awake during the procedure.
4	Laparoscopic surgery uses a camera to provide high-quality, magnified images of the surgical site, which can improve surgical precision and reduce the risk of complications.	Carbon dioxide used during pneumoperitoneum can be absorbed into the bloodstream, leading to carbon dioxide retention and acid-base disturbances, particularly in patients with impaired ventilation. Increased risk of venous thromboembolism: pneumoperitoneum can increase the risk of venous thromboembolism, particularly in patients with a history of blood clots or other risk factors [75].
5	Early return of functional status postoperatively. (Early return of bowel function post colectomy) [76].	Pneumoperitoneum can lead to insufflation-related injuries, such as puncture of blood vessels or organs, particularly if the procedure is performed by an inexperienced surgeon or if there is an underlying anatomical abnormality. Pneumoperitoneum-induced stretching of the parasympathetic nerve (vagus) can lead to bradycardia and subsequent hypotension.
6		Selective patient eligibility (lack of extensive intra-abdominal adhesions, tolerance of pneumoperitoneum)
Robotic		
1	Robotic-assisted surgery combines the advantages of laparoscopic surgery (less postoperative pain and faster recovery) with those of open surgery (high-quality three-dimensional view, restoration of eye-hand-target axis) [70].	Robotic surgery is often more expensive than traditional laparoscopic and open surgery due to the high cost of robotic technology and specialized training required for the surgeons and operating room staff [70].
2	Robotic surgery allows for greater precision and control of surgical instruments, which can be particularly important in complex surgeries.	Robotic surgery provides less tactile feedback to the surgeon than open surgery, which may impact surgical precision and increase the risk of complications.
3	Robotic surgery allows for a more ergonomic working environment for the surgeon, reducing the risk of fatigue and repetitive strain injuries.	Robotic surgery requires specialized training for surgeons and operating room staff, which can be time-consuming and may result in a steep learning curve [77].
4	The robotic arms used in robotic surgery are more dexterous than traditional laparoscopic instruments, allowing for more precise movements and greater surgical accuracy.	Robotic surgery may require greater reliance on robotic technology, which may reduce the surgeon's autonomy and control during the procedure.
5	Robotic instruments can move in a greater range of motion than traditional laparoscopic instruments, allowing for better access to hard-to-reach areas and more precise movements [78].	Limited use for instrumentation as each da Vinci instrument is limited to 10 uses [79].

Laparoscopic

Introduced in 1991, the laparoscopic CRC resection has been popularly used among surgeons across the globe due to its minimally invasive approach and relatively better outcome [51]. The laparoscopic approach is preferred over open surgery due to a multitude of advantages such as reduced intraoperative blood loss, shorter duration of postoperative ileus, improved pulmonary function, and reduced postoperative complications [52]. Despite the benefits, the laparoscopic method has a few drawbacks including a longer operative time, a higher rate of intraoperative complications, and bowel injury being the most reported [53,54].

Although 5 mm trocars don't prove to be associated with any significant morbidity, if placed oblique or imperfectly, it may lead to an entry site much larger than expected [55] which eventually results in more postoperative pain to the patient [56]. Conventional laparoscopy currently uses more than one 10-mm-sized trocars for optimum vessel management and is associated with a greater risk of complications, urging newer advancements with respect to colorectal surgeries to be made [55].

Single-Incision Laparoscopic Surgery

A rapidly developing approach which is a modified version of conventional laparoscopic surgery is the single-incision laparoscopic (SIL) surgery which allows for better cosmesis. With a single incision, the cosmetic outcome of the operation is much more preferred and may also have no visible scar if the incision is hidden within the umbilicus [57]. A single multichannel port site of size 3-5 cm is the only incision made on the abdomen [58] through which the entire surgery is carried out. Although only one incision is made, it is a relatively large defect created on the abdominal wall which may allow for wound site herniation [58]. On the other hand, the risk of wound site infections is low owing to the single incision site [57]. An article published by Maggiori et al. observed 8% of cases operated by SILS required to be converted to conventional multiparty laparoscopy and 2% needed to be converted to laparotomy [59].

Natural Orifice Transluminal Endoscopic Surgery

Making use of naturally occurring orifices, transanal, transvaginal, and transgastric, to gain access to the operative region, natural orifice transluminal endoscopic surgery (NOTES) has been promoted as a no visible scar approach [60]. With access gained through these natural orifices, a multitude of structures can be reached, including the distal rectum which is a challenge to be approached by conventional laparoscopic methods [60].

Although promising, this approach warrants a higher risk of perioperative infection due to contamination and improper access site closure [61]. The added complexity of extraction of resected tissue and the use of inadequate instruments make the approach even more difficult. Surgeons lose the advantage of being able to visually assess the viability of colonic tissue and securely place the stapler anvil [56]. Although many ideas for securing the stapler anvil in NOTES have been proposed, it has yet to convince the majority of surgeons [62]. Limited spatial orientation and current level of skills and instruments add to the issue and make learning this approach difficult [60].

Robotic Surgery

Introduced more than 20 years ago, robotic surgery was developed to overcome many of the major as well as minute drawbacks of laparoscopic surgery for an even more effective and ergonomically superior minimally invasive approach [63]. The robotic system has a console at a distance away from the surgical field, with which the surgeon operates a set of highly articulated instruments, emulating hand and wrist movements on the patient. This helps in eliminating the surgeon's tremors if any [64].

Unlike the laparoscopic approach, the perception of depth and three-dimensional visualization of structures in robotic surgery acts as an added advantage [65]. Like laparoscopy, robotic surgery also requires a specific skill set that needs to be learned but takes a shorter time to gain the said skill set with the help of simulators and computerized interfaces [64]. Accessing the deep pelvic region always has been a challenge, making rectal surgeries especially arduous in laparoscopy. The robotic approach makes it relatively much simpler to enter this area with depth visualization and slimmer arms that allow access into all quadrants of the abdomen without the need for intestinal repositioning [64]. The addition of other elements like fluorescent visualization and infrared technology to these robotic arms makes visualization even easier. One of the most consistently seen benefits of robotic surgery over laparoscopy is the lesser rate of operative conversions especially in cases of rectal cancer [66].

A meta-analysis published by Ng et al. observed significantly lower rates of surgical site infection in robotic-assisted resection in comparison to laparoscopic. Although intraoperative blood loss is observed to be less, operative time is significantly longer [67]. Despite being an ideal approach to colorectal resection, there are drawbacks, some of which include a long docking time that prolongs the surgery, the absence of tactile sensation, and a significantly higher cost, making its use difficult to justify especially in low-income areas (Table 1).

Prognosis

The prognosis of colon cancer depends on various factors including presenting complaints, its spread, molecular markers, and pathological staging. From all these, pathological stages at presentation are the most reliable indicators for prognosis. The most used pathological stages are Dukes' staging [80] and AJCC staging [81] (Table 2).

**Table 2:** CRC staging as per Dukes' and AJCC staging

	Dukes’ stage	AJCC/UICC stage
Tumor invading the submucosa or muscularis propria	A	I
Tumor invading through the muscularis propria	B	II
Evidence of tumor in regional lymph nodes	C	III
Distant metastasis	D	IV

The prognosis of stage 3 cancers depends upon T staging and nodal extension. The five-year survival rate of colon cancer according to the AJCC staging is given below [82]:

Stage I: 93%

Stage IIA: 85%

Stage IIB: 72%

Stage IIIA: 83%

Stage IIIB: 64%

Stage IIIC: 44%

According to this, early-stage tumors have good five-year survival rates [83]. The five-year survival rate is better in patients with EoCRC especially at ages 35 to 39 years than in late-onset at older ages [84]. Starting treatment within 60 days of diagnosis shows a good survival ratio [85]. Several other prognostic factors are being studied which include tumor markers at the most. Most commonly used are pretreatment CEA levels [86]. Carbohydrate antigen 19-9 (CA19-9), CYFRA 21-1, CA72-4, and Sialyl Lewis X antigen but these have low specificity and sensitivity [87,88].

There is also evidence of using Ras, transforming growth factor α and β-1, c-erbB2, c-myc, p53, deleted in colon cancer/allelic variance at 18q, p27, allelic loss/genome damage, and bcl-2 as histo-immunological prognostic markers [89]. Thymidylate synthase staining also has potential prognostic significance as TS is most targeted in chemotherapy. However, its significance in adjuvant therapy is still under study. Currently, the only marker with sufficient evidence to be used in routine practice is KRAS mutation and the selection for anti-epidermal growth factor receptor therapy [90]. Vascular endothelial growth factor, matrix metalloproteinases and inhibitors, Urokinase-type plasminogen activator and inhibitor, and Helix pomatia agglutinin are all under study for their role as prognostic indicators in CRCs [89]. Growing evidence also suggests that miRNAs play an important role in the metastatic process of CRC. miRNAs can be used as prognostic biomarkers to identify patients more likely to have micro-metastasis [91].

## Conclusions

Laparoscopic surgery is a well-established technique in CRC resection, but robotic surgery has become increasingly adopted for the same purpose. In our review, we tried to compare open vs laparoscopic vs robotic surgical techniques and the benefits and risks of each technique. Among the three techniques, minimally invasive surgery with laparoscopic and robotic surgery had significant improvement in short-term outcomes, but there was no significant difference found in overall disease-free survival rates, local recurrences, and distant metastasis incidence rates. Robotic surgery showed better perioperative clinical outcomes without compromising survival compared to laparoscopic surgery. Open surgeries are indicated more in complicated cases of colon cancer with perforation, obstruction, and hemorrhage.

Laparoscopic and robotic surgeries have a steep learning curve and sometimes have to be converted to open due to intraoperative complications. Currently, robotic surgeries are available only at a few specialized centers and involve longer operative time and higher costs compared to open and laparoscopic approaches, but it is feasible and safe compared to conventional surgery. Robotic surgeries have better short-term outcomes and similar rates of long-term postoperative complications. Additional well-defined, multi-centered randomized control trials are required to further validate robotic surgery over open and laparoscopic approaches.
